# Spatiotemporal patterns and implications of suspended *Alexandrium catenella* cysts in the Pacific Arctic region

**DOI:** 10.1016/j.dsr2.2025.105567

**Published:** 2025-11-27

**Authors:** Evangeline Fachon, Robert S. Pickart, Jie Huang, Catherine Lalande, Donald M. Anderson

**Affiliations:** a Biology Department, Woods Hole Oceanographic Institution, 266 Woods Hole Road, Woods Hole, MA, USA; b Massachusetts Institute of Technology (MIT)-WHOI Joint Program in Oceanography/Applied Ocean Science & Engineering, Woods Hole, Cambridge, MA, USA; c Physical Oceanography Department, Woods Hole Oceanographic Institution, 266 Woods Hole Road, Woods Hole, MA, USA; d Korea Polar Research Institute, Division of Ocean and Atmosphere Sciences, Songdomirae-ro 26, Yeonsu-gu, Incheon, South Korea

**Keywords:** Harmful algal blooms, HABs, *Alexandrium catenella*, Pacific Arctic region, Resting cyst, Resuspension

## Abstract

The persistent presence of a massive accumulation of resting cysts of the toxic dinoflagellate *Alexandrium catenella* on the Chukchi shelf represents a significant risk to Pacific Arctic ecosystems, as warming conditions are likely to promote harmful algal blooms of this species. While the majority of cysts are found in the benthos, cysts can also be suspended through the water column, allowing them to be transported by currents or to experience elevated temperature and light conditions that may accelerate germination. Spatial patterns of cyst suspension were investigated during a Fall 2020 survey, revealing broad presence of suspended cysts in near-bottom waters overlying benthic cyst accumulations. Enhanced suspension was observed at shallow, well-mixed stations – in some places extending beyond the bottom boundary layer into surface waters. Sinking particles collected continuously from 2017 to 2019 showed seasonal patterns of cyst flux, with export pulses during the late summer and wind-driven resuspension peaking during the fall. To evaluate the potential contribution of resuspension to bloom initiation, a hydrographic climatology of the southern Chukchi Sea was used to estimate cyst germination rates through the water column during the summer (June–September). This analysis was paired with wind-driven 1-D mixing simulations, demonstrating that cyst access to surface waters is enhanced under stormy conditions. While storm events are not currently common during the summer bloom season, a trend towards increasingly windy conditions points towards future potential for resuspended cysts to contribute to blooms in the shallow and warming waters of the Pacific Arctic region.

## 1. Introduction

As water temperatures warm and sea ice thins and decreases in extent, phytoplankton in the Pacific Arctic region are experiencing expanded spatiotemporal opportunities for growth (Arrigo et al., 2008, 2014; Kahru et al., 2011). These changing conditions are driving widespread concern about the emergence of harmful algal blooms (HABs) as a threat to polar ecosystems and human health (Anderson et al., 2022; Fachon et al., 2025; Huntington et al., 2020; Lefebvre et al., 2022). In particular, the dinoflagellate *Alexandrium catenella* produces a suite of toxins, collectively known as paralytic shellfish toxins (PSTs), which are responsible for paralytic shellfish poisoning (PSP). PSTs accumulate in the tissues of organisms that consume *A. catenella*, such as bivalves, zooplankton, or small fish; these toxins are subsequently transferred through the food web (Oyaneder Terrazas et al., 2017) and have been detected widely in marine birds and mammals in Pacific Arctic waters (Lefebvre et al., 2016; Van Hemert et al., 2021).

**Fig. 1.**
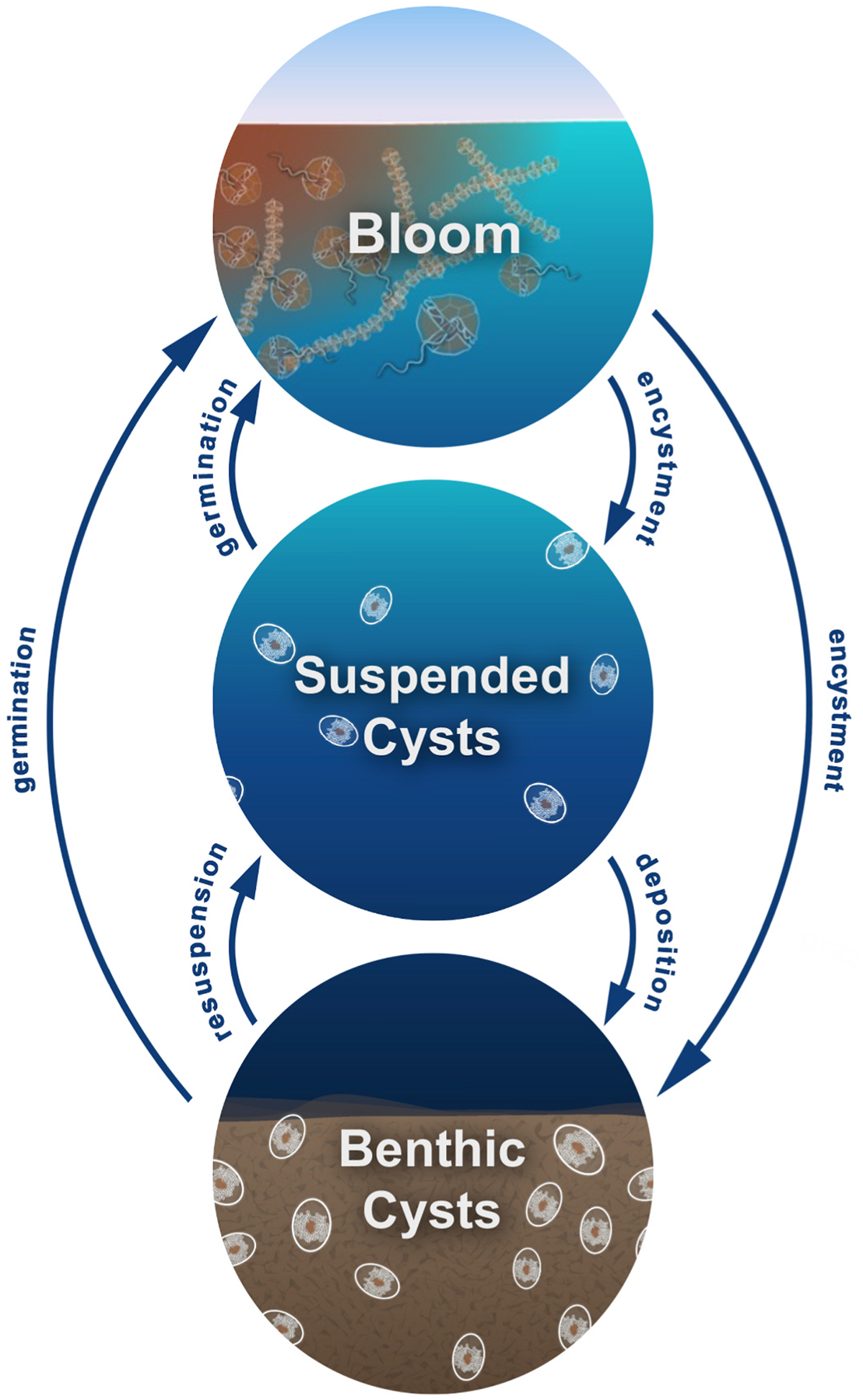
Conceptual diagram illustrating the connections between *Alexandrium* groups considered in this study (benthic cysts, suspended cysts, and blooming vegetative cells). Benthic cysts may be resuspended by physical (currents, tidal energy, storms) or biological (bioturbation, e.g. Shull et al., 2014) forces. Once resuspended, cysts may germinate if conditions are appropriate, or they will return to the cyst bed once conditions are conducive to deposition. New cysts produced by vegetative populations (encystment) may form in suspension, or near-bottom where they directly contribute to the benthic cyst population (Brosnahan et al., 2015). At all times, *Alexandrium* cyst and vegetative cell distributions are influenced by additional processes such as transport, grazing, bioturbation and death. (Adapted from schematic by Natalie Renier, WHOI Creative Studio).

*Alexandrium catenella* is a globally distributed species, with blooms occurring in both hemispheres and across a range of latitudes (Mons et al., 1998). We note that this algae has been previously referred to in the region as *Gonyaulax tamarensis* (Bursa, 1963)*, A. fundyense* (Natsuike et al., 2017)*, A. tamarense* (Natsuike et al., 2013), and Atama complex (Group 1) (Gu et al., 2013), prior to a taxonomic revision (John et al., 2014) which identifies the species as *A. catenella* based on an updated molecular phylogeny rather than morphological criteria previously used. The ubiquitous presence of *A. catenella* can be partially attributed to its meroplanktonic life cycle, which alternates between free-swimming bloom-forming cells and resting benthic cysts (Fig. 1). This benthic cyst stage can survive for years to decades, promoting survival through adverse conditions and allowing germination to occur when temperature and oxygen concentration are favorable (Anderson et al., 2005). Germination is mediated by dormancy cycling: resting cysts cycle through periods of dormancy and quiescence (Fischer et al., 2018; Brosnahan et al., 2020), with quiescent cysts primed to germinate under favorable conditions. The interaction between annual temperature trends and dormancy cycling shapes seasonal patterns of bloom occurrence (Brosnahan et al., 2020). In some areas cysts can accumulate in the sediment, and these high-density regions, known as cyst beds, serve as a source for future blooms (Anderson et al., 2014).

The resilient properties of resting cysts have allowed *A. catenella* to carve out a substantial foothold in the Pacific Arctic region; cyst presence was first reported on the Chukchi shelf in 2013 (Gu et al., 2013) in some of the highest concentrations ever recorded globally (Natsuike et al., 2013). Subsequent investigation by Anderson et al. (2021) revealed an extensive cyst bed, unprecedented in geographic scale at over 100,000 km^2^. The cyst bed extends north from the Bering Strait across the Chukchi shelf, with a smaller accumulation in the western Beaufort Sea, collectively representing a massive reservoir of bloom potential (Anderson et al., 2021). The long-term history of *A. catenella* in the region is uncertain; while there have been sporadic reports of blooms over the last half-century (Bursa, 1963; Walsh et al., 2011), the majority of observations come from recent years (Einarsson et al., 2022; Fachon et al., 2025; Natsuike et al., 2017a). This lack of consistent historical observations poses challenges in determining whether the increase in recent bloom records represents a true emerging trend, but it is clear that warming in the Chukchi Sea, which has accelerated in recent years (Danielson et al., 2020), can facilitate germination of cysts in areas that were previously unfavorable for growth (Anderson et al., 2021; Lago et al., 2025).

While mapping efforts have focused on benthic cyst abundance, resuspension and overall prevalence of cysts in the overlying waters have yet to be characterized in the Pacific Arctic region. In the Gulf of Maine, where large *Alexandrium* blooms and subsequent fisheries closures occur annually, significant numbers of cysts are found suspended in and above the benthic nepheloid layer (Pilskaln et al., 2014a, 2014b), a high-turbidity region that is part of the bottom boundary layer (BBL). The BBL is the region of the water column that is influenced by the sediment-water interface (Boudreau and Jorgensen, 2001), and turbulence within the BBL can mobilize and transport sediments and other benthic materials (Hill and McCave, 2001). Cysts suspended within and above the BBL can experience elevated sunlight, temperature, and oxygenation relative to their benthic counterparts, which could greatly enhance germination potential if suspension corresponds with intervals when the cysts are quiescent. While *A. catenella* cysts can germinate in darkness, their rate of germination increases significantly in the presence of light (Anderson et al., 2005). Although the majority of cysts are found in dark and turbid bottom waters, sufficient mixing, such as through storm activity, can resuspend and deliver them to the mid and upper water column. In a comparison of benthic and suspended cyst abundances in the Gulf of Maine, it was estimated that even low levels of suspension could result in water-column germination that rivals benthic germination (Kirn et al., 2005).

Factors that govern cyst resuspension are varied and dynamic, introducing complexity into the interpretation or prediction of resuspension patterns. Cysts and other particles can be resuspended by large-scale mixing events such as storms, but also through tidal or current energy when near-bottom velocities are sufficient to cause erosion (Butman et al., 2014). Once resuspended, residence time in the water column is determined by settling rate, which is in turn determined by the properties of the individual cyst (Anderson et al., 1985) as well as the characteristics of any aggregations or flocs that the cysts comprise. As the cysts settle, they are transported by currents, and repeated resuspension events can carry cysts long distances from their initial site of deposition (Aretxabaleta et al., 2014). Beyond resuspension of mature cysts, new cysts are delivered to the system when blooms terminate and planozygotes encyst (Brosnahan et al., 2015). In the Arctic, the timing of these settlement pulses is not well understood.

This study employs a combination of observational analysis and modeling to investigate spatial and seasonal patterns of *A. catenella* cyst suspension in the Pacific Arctic region, and to characterize the potential role that resuspended cysts may play in bloom dynamics. During a hydrographic survey conducted on the Chukchi and Beaufort shelves in fall 2020, paired water and sediment samples were collected to evaluate spatial patterns of suspended cyst concentration. To examine how suspended cyst concentrations change over an annual cycle, cyst fluxes were measured in three sediment traps deployed at Distributed Biological Observatory (DBO) locations from 2017 to 2019 (Lalande et al., 2021; O’Daly et al., 2020). A hydrographic climatology from the southern Chukchi shelf was used to estimate environmental suitability for cyst germination over the course of the summer (June–September) based on seasonal light and temperature conditions through the water column. Finally, wind events of varying intensity were used in conjunction with a mixing model in order to understand the conditions needed for suspended cysts to mix into surface waters. These simulations were evaluated against historical wind data to assess the potential frequency of such events in the region, and future scenarios are considered.

## 2. Materials and methods

### 2.1. Alexandrium catenella cyst survey

**Fig. 2.**
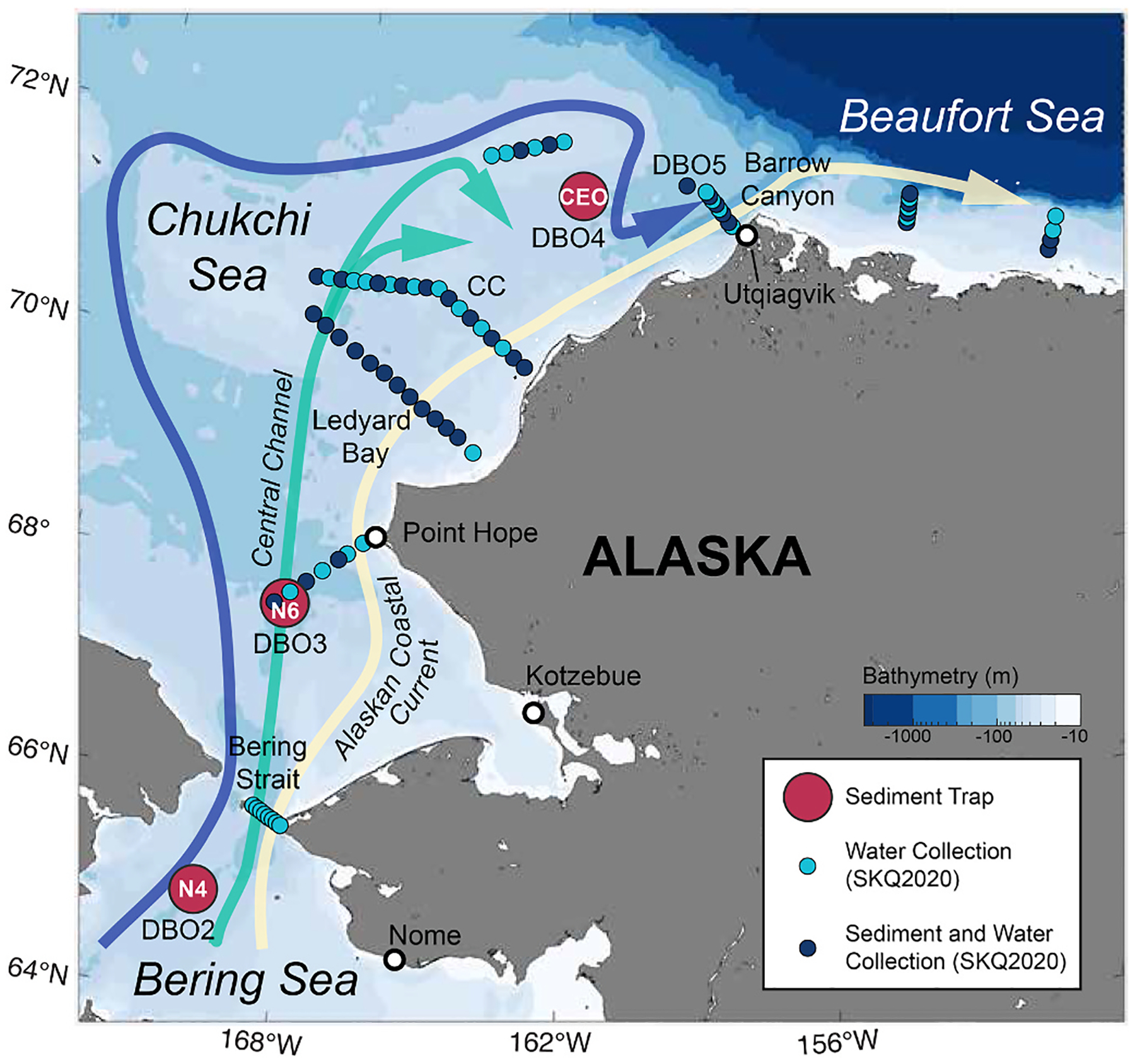
Map of the study region with stations sampled during SKQ2020_14S (October–November 2020) and sediment trap locations. Arrows indicate generalized major regional flow pathways (after Corlett and Pickart, 2017) including the Central Channel Branch and the Alaskan Coastal Current. The bathymetry is from ETOPO-2.

A regional survey of benthic and suspended *A. catenella* cyst distribution was conducted aboard the R/V *Sikuliaq* from 15 October to 19 November 2020 (SKQ2020_14S) in the Chukchi and Beaufort Seas (Fig. 2). At each station, the water column was profiled using a Sea-Bird 911plus conductivity-temperature-depth (CTD) instrument mounted on a 24-place rosette with 12 L Niskin bottles. The package contained additional sensors to measure oxygen, fluorescence, and transmissivity, among other variables (McRaven and Pickart, 2024). The CTD sensors were calibrated at Sea-Bird prior to the cruise, and the conductivity underwent an additional in-situ calibration using deep water samples collected seaward of the shelf. Final downcast 1-db pressure-averaged profiles were produced using Sea-Bird processing scripts. The resulting accuracies for temperature and practical salinity are estimated to be 0.002 °C and 0.003, respectively.

Samples for suspended cyst abundance analyses were harvested from the Niskin bottles mounted on the CTD rosette. At most stations, bottom water was collected from the deepest point of the CTD cast, approximately 1–2 m above the seafloor depending on swell conditions. Along the Ledyard Bay transect, additional samples were collected ~10 m above seafloor and 2–3 m below the surface. For each sample, 12–24 L of seawater was concentrated directly from the Niskin through a 20 μm mesh sieve, which was then backwashed to 15 mL and preserved in 5 % formalin. Within 72 h of collection, these samples were centrifuged (10 min at 3000×*g*), supernatant was aspirated, and the resulting pellets were resuspended in methanol and stored frozen (−20 °C). At selected locations on the shelf (n = 41), sediment samples were collected via a 0.1-m^2^ van Veen grab. A subsample of the surface 0–3 cm layer from each grab was homogenized and stored in the dark (4 °C) for the duration of the cruise.

Spearman’s rank correlations were calculated to investigate the relationship between cyst densities and various environmental parameters. A linear interpolation between sampling points was used to estimate the relative number of cysts present throughout the water column and in the benthos along the Ledyard Bay transect.

### 2.2. Moored sediment traps

Sequential sediment traps (24 cups, Hydro-Bios, Germany) were deployed south of the Bering Strait at the DBO2 site (N4; 64°55 N, 169°55 W) and north of the Bering Strait at the DBO3 site (N6; 67°40 N, 168°44 W) from June 2017 to June 2019, as well as on the northern Chukchi shelf at the DBO4 site (Chukchi Ecosystem Observatory; 71°35 N, 161°31 W) from August 2018 to July 2019 (Fig. 2) (Lalande et al., 2021). Water depth at each site ranged from 46 to 50 m, and the sediment traps were deployed 7–15 m above the seafloor. Trap cups were programmed to rotate at intervals ranging from one week to one month, and each cup was filled with seawater adjusted to a salinity of 38 and fixed with 4 % formalin to preserve the sample. Upon retrieval, samples were analyzed for zooplankton and meroplankton, chlorophyll-a, algal cells, total particulate matter (TPM), and particulate organic carbon (POC) (see Lalande et al., 2021 for details). Subsamples from each trap were processed for dinoflagellate cyst identification and enumeration following the methods outlined in section 2.3. Cyst fluxes were converted to cyst flux cm^−2^ d^−1^ and annual cyst fluxes were calculated by integrating the daily fluxes across the entire deployment and normalizing to 365 days. To investigate the relationship between cyst deposition and wind, time series of averaged wind speeds around each mooring (0.5 × 0.5-degree box) were calculated from the ERA5 Reanalysis (Hersbach et al., 2020).

### 2.3. Primuline staining and microscopy

All samples were processed for cyst abundance following protocols outlined in Anderson et al. (2014) and Yamaguchi et al. (1995), with some modifications based on sample type. Homogenized surface sediments were subsampled (2 cc), diluted in seawater, and sonicated (Branson Sonifier 250, 0.5-inch probe) for 1 min at 40 % amplitude. The sonicated sediments were sieved to isolate the 20–80 μm size fraction and resuspended in seawater fixed with 5 % formalin. Sediment trap samples were similarly processed using 2 mL of homogenized slurry from each trap sample. Formalin-fixed samples were centrifuged (10 min at 3000×*g*), and supernatant was aspirated, replaced with methanol, and maintained at −20 °C for at least 72 h. A series of centrifugation steps were used to remove the methanol, rinse the samples with deionized (DI) water, and resuspend in 2 ml primuline (2 mg ml^−1^). Samples were rotated for 1 h in the dark at 4 °C on a Labquake rotator, and then an additional series of centrifugation steps was used to rinse the samples with DI water and resuspend them to a final volume of 2–15 mL (determined by sample density) for microscopy.

A homogenized aliquot from each sample was loaded into a Sedgewick-rafter counting chamber and scanned on a fluorescent microscope at 10 × magnification under a FITC filter (Zeiss 09, excitation = [450–490] nm BP; emission = [515] nm LP). Intact *A. catenella* cysts identified by their distinctive oblong shape and internal contents were enumerated and converted to cysts cm^−3^ for sediment samples, cysts m^−3^ for water samples, and cysts cm^−2^ d^−1^ for sediment trap samples.

### 2.4. Modeling mixing and germination potential

To explore the potential interaction between cyst resuspension and germination, a climatology of hydrographic profiles was used to investigate the likelihood of mixing and resuspension during the summer bloom season (June–September), as well as to model germination potential across different levels of the water column through the summer. A compilation of 476 hydrographic profiles used for these analyses were collected along the DBO3 line from 2002 to 2019 during the summer months (see Pickart et al., 2023 for details). Supplementary Fig. 1 shows the temporal distribution of occupations used in this study. For each occupation, the height of the BBL was defined using the CTD profiles of density, salinity, and temperature following the methodology of Pickart et al. (2002).

#### 2.4.1. Modeling germination

Hydrographic temperature profiles and seasonal light conditions were used to estimate environmental suitability for cyst germination through the water column based on the DBO3 climatology. Parameters and equations for germination (Supplementary Table 1) were acquired from previous studies (Anderson et al., 2005; McGillicuddy et al., 2005) used to model population dynamics in the Gulf of Maine. For the vertical range of each profile, the fraction of a quiescent cyst population likely to germinate per day (% population d^−1^) was calculated at regular depth intervals based on temperature and depth-attenuated photosynthetically active radiation (PAR) (W m^−2^) following the relationships defined in Anderson et al., (2005) and irradiance climatology reported in Dissing and Wendler (1998) (see supplementary methods for equations). Profiles were aggregated by month (June–September), and germination potential was estimated for the bottom, averaged within the BBL, and averaged within the upper water column (all depths above the BBL) of each profile.

#### 2.4.2. Frequency of wind events and modeling of the mixed layer depth

Historical wind velocity data (1979–2022) for the southern Chukchi Sea on the DBO3 line were obtained from the ERA5 Reanalysis (Hersbach et al., 2020), focusing on the months of June–September. Within this time frame, peaks in wind speed were identified, and composite wind event profiles were constructed by grouping peaks of varying intensity and averaging wind speeds over the 3-day window around each peak. The average frequency of wind events in each month was calculated by identifying peaks in the ERA5 dataset and averaging their occurrence across years. Extreme years were also characterized by the maximum number of wind events in a given month within the dataset. Additionally, historical summer (June–August) wind speeds were obtained from the ERA5 Reanalysis over the Ledyard Bay cyst bed.

The Price et al. (1986) 1-D mixing model (hereafter referred to as PWP) was used to investigate the effect of wind on the surface mixed-layer depth using the hydrographic profiles from the DBO3 climatology. In each simulation, a hydrographic profile was used to represent initial conditions, and wind stress was calculated and applied in the form of repeated 3-day wind events of defined peak intensity. This simulation was iterated on the same initial hydrographic profile using different peak wind scenarios (e.g. 10 m s^−1^, 12 m s^−1^, 14 m s^−1^, 16 m s^−1^, 18 m s^−1^), and the depth of the mixed layer was documented through time under each forcing scenario. This process was repeated on a subset of 14 representative hydrographic profiles from the climatology. Each representative profile was obtained by averaging various historical profiles with similar bottom and BBL depths. The output of these simulations is used to evaluate how many wind events of a given strength would be needed for the surface mixed layer to access regional BBLs, providing suspended cysts the opportunity to be mixed into surface waters.

## 3. Results

### 3.1. Spatial distribution of benthic and suspended cysts

**Fig. 3.**
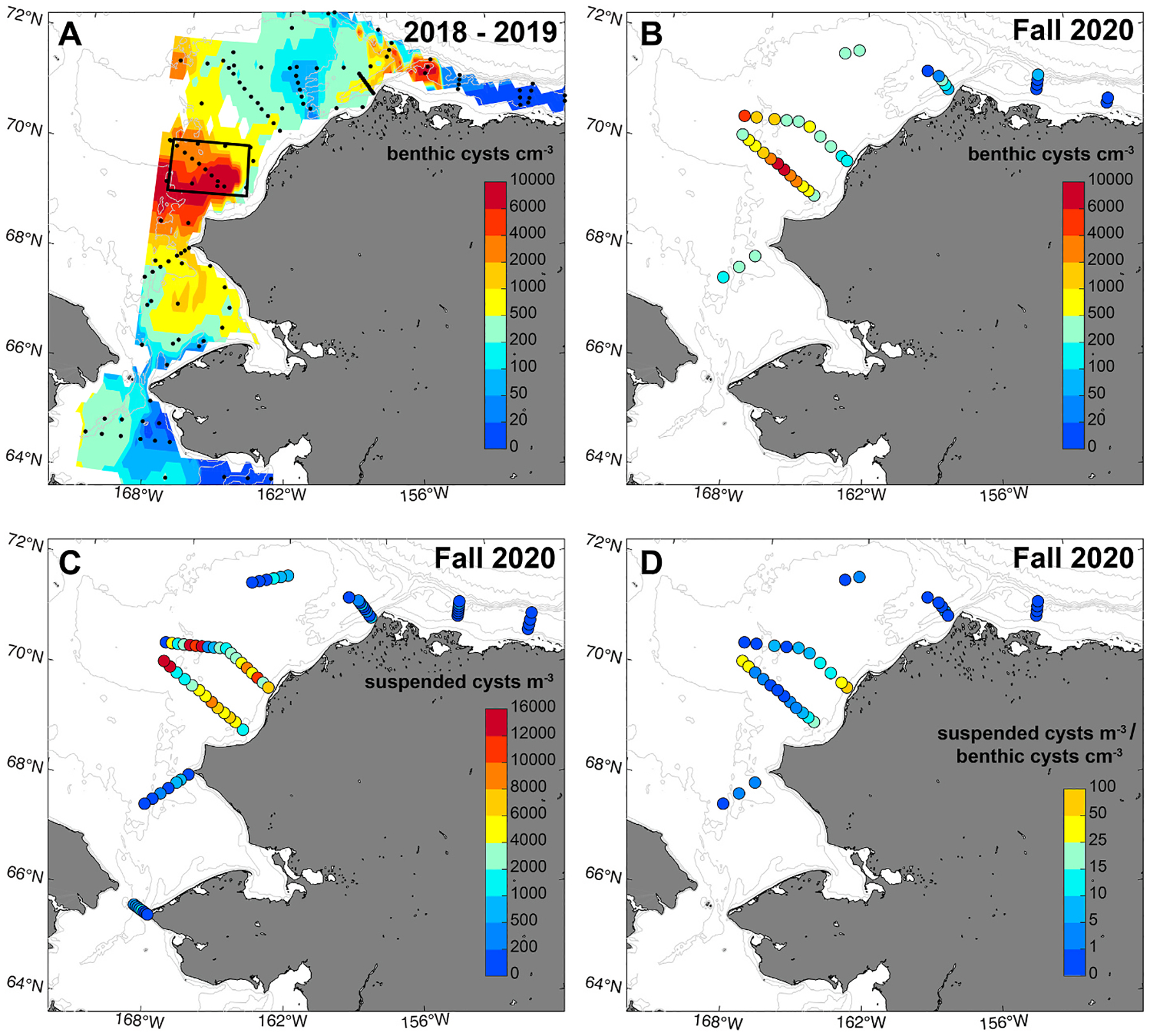
*A. catenella* cyst concentrations (A) in the 0–3 cm layer of the benthos based on a compilation of several regional surveys conducted in 2018–2019 (reproduced from Anderson et al., 2021), (B) in the 0–3 cm layer of the benthos during the SKQ2020_14S survey (October–November 2020), (C) in the near-bottom waters measured during the SKQ2020_14S survey (October–November 2020), and (D) Ratio of near-bottom suspended to benthic cysts at all sites where both metrics were collected. The black box in A indicates the area of the Ledyard Bay region considered for the wind analysis (Fig. 8).

Suspended *A. catenella* cysts were found widely in near-bottom waters throughout the region of the Chukchi shelf *A. catenella* cyst bed, although there were noticeable differences in the distribution patterns of benthic and suspended cysts (Fig. 3). Overall, benthic cyst levels observed in the fall 2020 survey were consistent with data collected in previous years (Fig. 3A), with highest concentrations (8600 cysts cm^−3^) observed in the central Ledyard Bay region (Fig. 3B). By contrast, the greatest suspended cyst concentrations (15,800 cysts m^−3^) were observed on the offshore side of the Ledyard Bay transect (Fig. 3C). Both the Ledyard Bay and Central Channel transects showed elevated presence of suspended cysts at inshore and offshore stations, with lower concentrations in the middle of each transect. This distribution of suspended cysts appears to correspond with major transport pathways in the area; the Alaska Coastal Current runs along the coast while the Central Channel pathway crosses the offshore region of the study area (Fig. 2). However, at the time of sampling there was no correlation between near-bottom absolute geostrophic velocity and cyst resuspension (Table 1). Outside of the main cyst bed area, suspended cysts were found at relatively low levels in the Bering Strait and Chukchi Sea and were nearly absent at stations along the Beaufort Shelf.

Distinct spatial distributions of benthic and suspended cysts indicate that other factors beyond benthic cyst concentrations are driving patterns of resuspension. To better understand these differences, a ratio was calculated between near-bottom suspended and benthic cyst concentrations (Suspended cysts m^−3^: Benthic cysts cm^−3^) for all stations where paired data were collected and cysts were present (n = 36, Fig. 3D). In some cases, no suspended cysts were recorded even when cysts were present in the underlying benthos. Spearman’s rank correlation was used to evaluate the relationship between benthic cyst concentration, suspended cyst concentration, suspended:benthic ratio, and various co-measured metrics (Table 1, Supplementary Fig. 2). Beam transmission, a measurement used to assess water clarity, was negatively correlated with all cyst metrics, indicating that higher overall particulate loading was associated with greater benthic and suspended cyst concentrations. In particular, when beam transmission was <50 %, suspended cysts were much more prevalent in near-bottom waters (Supplementary Fig. 2A). Near-bottom fluorescence, an additional indicator of overall sediment resuspension (Pirtle-Levy et al., 2009), was positively correlated with all cyst metrics. Cyst suspension was more pronounced at shallow shelf stations and reduced sharply with increasing water depth (Supplementary Fig. 2C). Additionally, the suspended:benthic ratio was positively correlated with oxygen and negatively correlated with salinity.

### 3.2. Vertical cyst resuspension

**Fig. 4.**
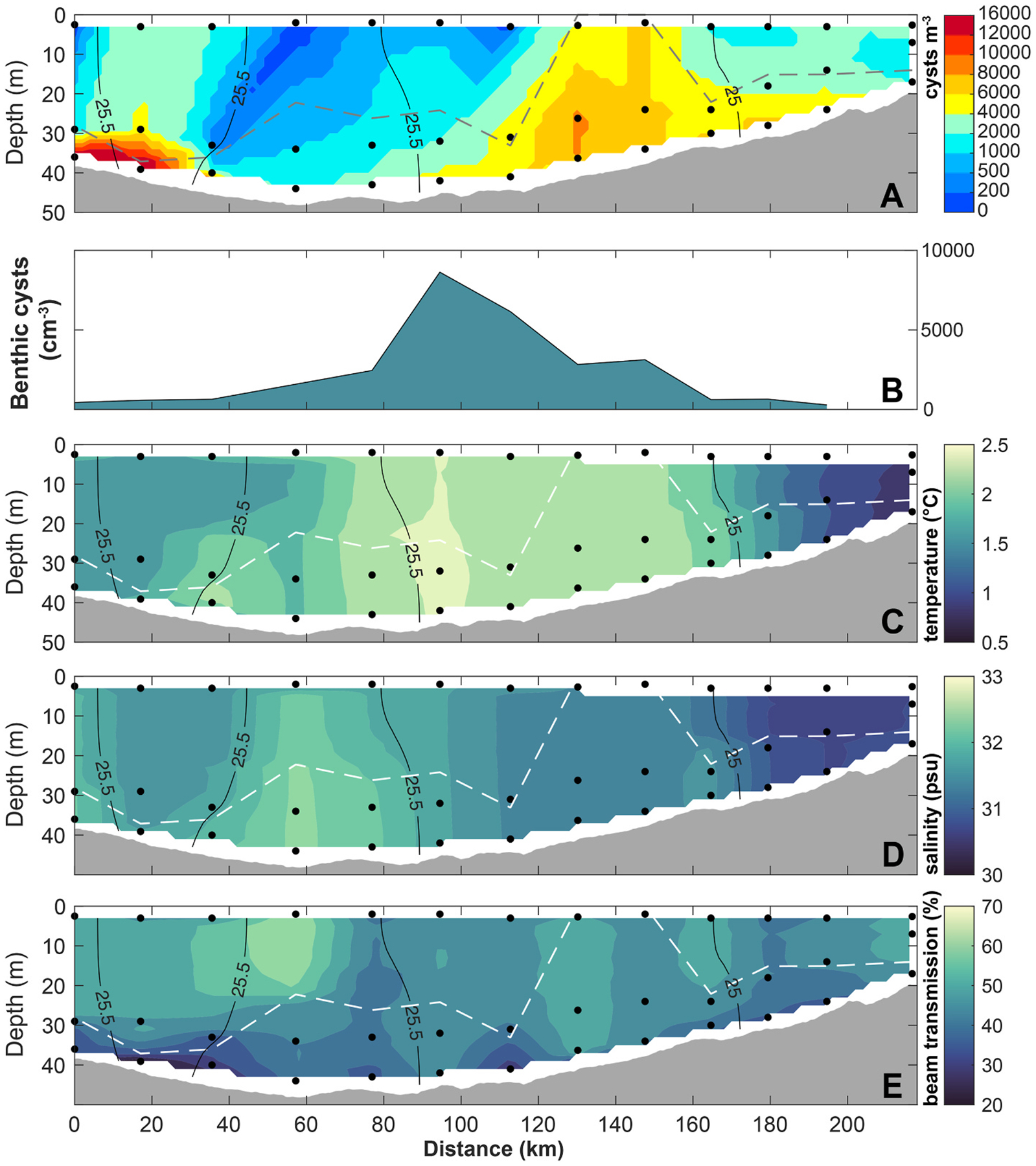
(A) Vertical section of *A. catenella* cyst concentration along the Ledyard Bay transect and (B) underlying benthic cyst concentrations at each station (November 2020). Hydrographic sections of (C) temperature, (D) salinity, (E) beam transmission, are displayed across the transect. Black contours are potential density (kg m^−3^). The dashed line indicates the height of the BBL at each station. Black points mark the locations of discrete water samples collected for cyst abundance analysis. Station distance is indicated from the offshore (0 km) to the onshore (217 km) side of the transect.

Along the Ledyard Bay transect, the epicenter of the Chukchi cyst bed, suspended *A. catenella* cysts were prevalent within and above the BBL (Fig. 4A). While maximum suspended cyst concentrations consistently occurred near the seafloor, cysts were detected in 10 out of 13 (77 %) surface samples. Similar to the spatial distribution observed in near-bottom waters, surface cyst concentrations were notably higher near the mid-inshore portion of the transect (up to 6500 cysts m^−3^) at stations where the water column was well-mixed, with weak stratification in temperature (Fig. 4C) and salinity (Fig. 4D). The average surface and near-bottom suspended cyst concentrations across the Ledyard Bay transect were 2200 ± 2000 cysts m^−3^ and 6200 ± 4600 cysts m^−3^ respectively. When all results were interpolated across the Ledyard Bay transect, the quantity of cysts present in suspension along the transect was orders of magnitude lower than the reservoir of cysts in the top 1 cm of the benthos, with suspended cysts representing only ~0.45 % of the total cyst population calculated per unit area. If we consider that only the top mm of the benthic population is able to germinate (Ishikawa et al., 2014; Kirn et al., 2005), suspended cysts represent ~4.4 % of the cyst population with access to the water column during this survey; similar results have been calculated for *A. catenella* cyst populations in the Gulf of Maine (Kirn et al., 2005).

### 3.3. Cyst fluxes

**Fig. 5.**
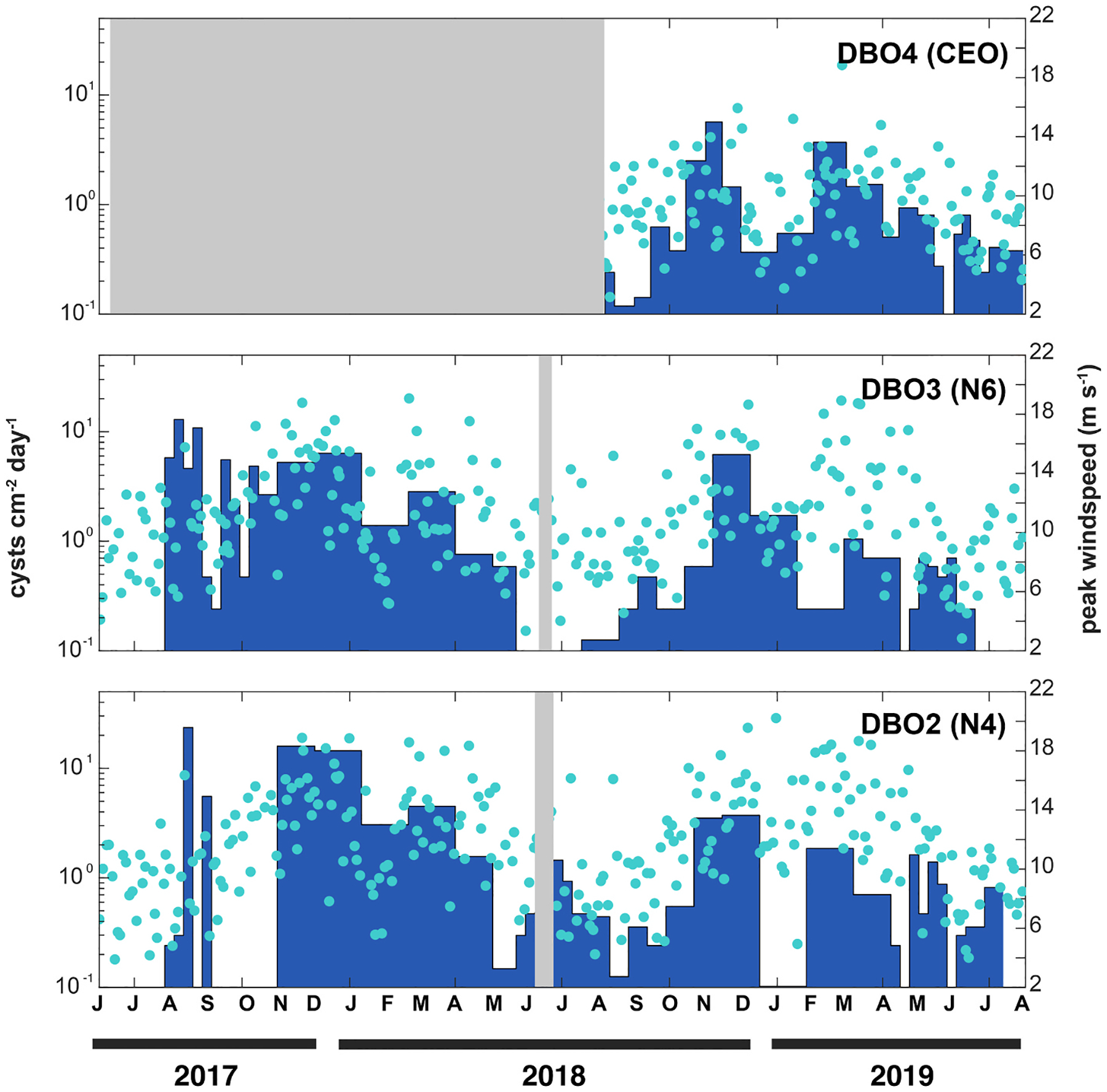
*A. catenella* cyst fluxes in sediment traps (bars) from June 2017 to July 2019 for DBO4 (top), DBO3 (middle), and DBO2 (bottom); note that fluxes are plotted on a log scale (left axis). Wind speed (right axis) and timing of peak wind events are plotted as points. Wind speeds from the ERA5 Reanalysis were averaged over a 0.5 × 0.5° box around each sediment trap site for the span of the deployments. Boxes indicate periods without data, note that the DBO4 mooring was only deployed in 2018–2019.

Downward fluxes of *A. catenella* cysts 7–15 m above the seafloor varied spatially, seasonally, and interannually in the Pacific Arctic region (Fig. 5). While cyst fluxes varied over several orders of magnitude across the dataset, cysts were present throughout the year and found in 80 % of the sediment trap samples. Cyst fluxes were positively correlated with the peak wind speed and this correlation was strongest when peak winds in the 4 days leading up to each sampling interval were considered in addition to wind conditions during the sampling interval itself (Spearman’s rank correlation, rho = 0.42, p < 0.001). This offset accounts for the lag between disturbance and deposition, as it may take several days for cysts to resettle following suspension.

Peak wind speeds increased in the fall and winter (Fig. 5) and were associated with higher levels of cyst flux into the sediment traps relative to the spring and summer. However, we note that the largest fluxes at the DBO3 site occurred under moderate peak wind conditions (11–13 m s^−1^) in August 2017 (max 12.9 cysts cm^−2^ d^−1^, Table 2). This is around the same time that maximum cyst fluxes were recorded at the DBO2 site (23.5 cysts cm^−2^ d^−1^, Table 2), although this sampling interval was associated with an anomalous summer wind event (16 m s^−1^). Based on the timing and magnitude of the August fluxes at both sites, and the scarcity of cysts in the sediment traps just prior, we believe that these pulses may represent recent encystment and deposition from a terminating bloom event rather than settlement of a resuspended population. Cyst fluxes at both DBO2 and DBO3 sites in the fall and winter of 2018–2019 were lower than during the previous year. Cyst fluxes collected at the DBO4 site during 2018–2019 were similar in magnitude to the southern sites, with the highest fluxes (5.7 cysts cm^−2^ d^1^) recorded in November 2018 (14 m s^−1^ peak winds), and a secondary peak observed in February 2019 (19 m s^−1^ peak winds).

Annual cyst fluxes ranged from 356 to 1772 cm^−2^ yr^−1^ and were highest at the DBO2 and DBO3 sites during the 2017–2018 deployment cycle. Notably, these values were generally greater than the average surface cyst concentrations recorded in each region following the trap deployment (Table 2, Fachon and Anderson, 2021). For example, the integrated cyst flux at DBO2 for the 2017–2018 trap deployment was 1772 cysts cm^−2^ year^−1^, but in summer of 2018 cyst concentrations in the underlying sediments were much lower (196 ± 164 cysts cm^−3^). Similar results were observed at DBO3 for the same period, with an integrated flux of 1078 cysts cm^−2^ year^−1^ and an underlying concentration of 399 ± 218 cysts cm^−3^ in the benthos.

### 3.4. Seasonality of wind events, hydrography, and germination potential

#### 3.4.1. Germination potential

**Fig. 6.**
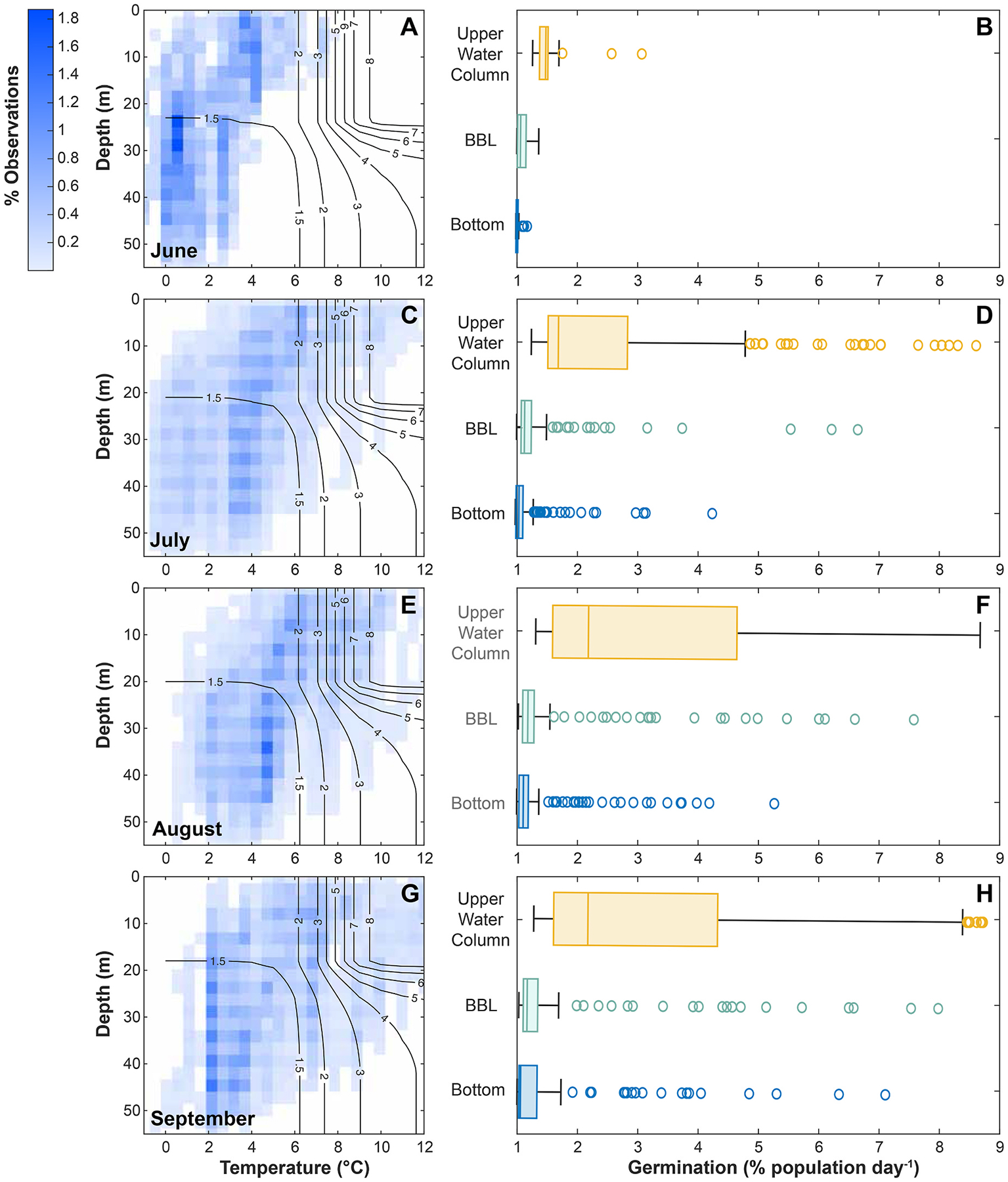
Heat maps of suitability for *A. catenella* cyst germination throughout the water column using the hydrographic climatology from DBO3, based on temperature and estimated light availability across depths for each summer month: (A) June, (C) July, (E) August, (G) September. Contours in each panel represent germination rates (% population day^−1^) predicted by equations presented in Anderson et al., 2005; McGillicuddy et al., 2005 (see Supplementary Methods). For each profile in the climatology (n = 476), germination rate at various levels of resuspension was estimated by calculating germination for the bottom (benthic cysts), averaging through the BBL (resuspended cysts confined to the BBL), and averaging through the upper water column (resuspended cysts above the BBL). Distribution of these rates in the three levels of the water column is displayed in boxplots for each month (B) June, (D) July, (F) August, (H) September.

Germination potential increases throughout the summer at all levels of the water column to varying degrees, with the strongest change occurring in the upper water column (Fig. 6). For benthic cysts, the estimated germination rate increased from an average 1.03 ± 0.04 % d^−1^ in June (Fig. 6B) to 1.63 ± 1.24 % d^−1^ in September (Fig. 6H). In the BBL, estimated germination rate increased from an average 1.10 ± 0.10 % d^−1^ in June to 1.87 ± 1.61 % d^−1^ in September. The upper water column had the highest estimated germination rates as well as the greatest range in germination, with an average of 1.51 ± 0.32 % d^−1^ in June to 3.36 ± 2.46 % d^−1^ in September.

#### 3.4.2. Frequency of wind events and effects on surface mixed layer depth

**Fig. 7.**
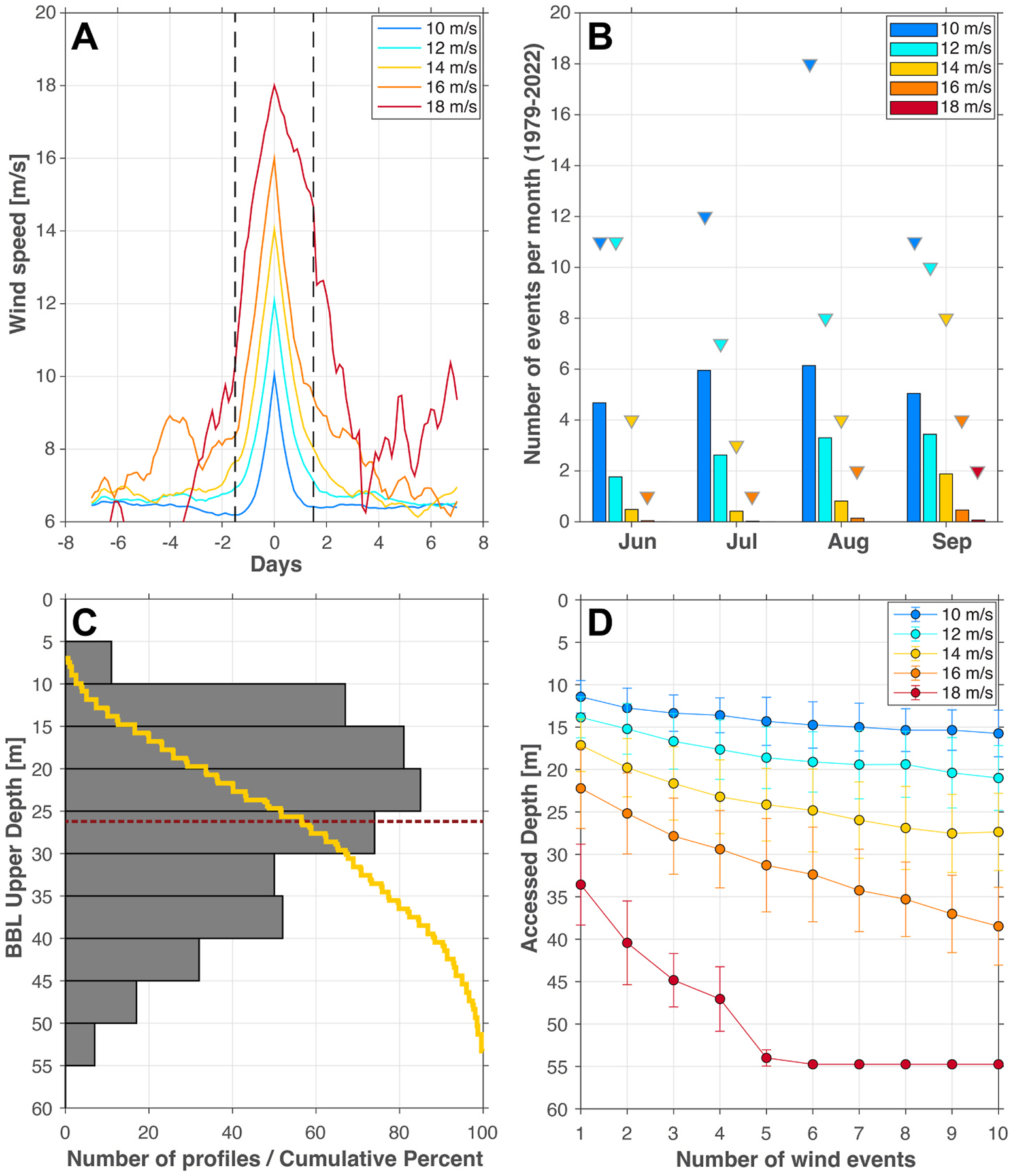
Frequency of summer wind events and their effect on mixed layer depth. (A) Composite wind events of varying peak intensity (10–18 m s^−1^) based on the ERA5 climatology (1979–2022) from the southern Chukchi Sea along the DBO3 line. The black dashed lines indicate a 3-day window from 1.5 days before peak wind to 1.5 days after peak wind. (B) Number of wind events per month delineated by strength (color), with mean occurrence displayed by the bar graph and maximum occurrence indicated by the triangles. (C) Histogram of upper depths of the BBL across all hydrographic profiles in the DBO3 climatology; the mean is indicated by the red dashed line and the yellow line displays the cumulative distribution. (D) Depth of the water column accessed by the surface mixed layer via repeated wind events of varying intensity (color), using the PWP model (see text for details). Circles represent the mean and bars represent the standard deviation across all hydrographic profiles which were used as initial conditions for the model run (n = 14).

On average, the frequency and intensity of wind events in the study region escalates over the course of the summer, with strong and repeated wind events facilitating enhanced mixing between the BBL and surface waters (Fig. 7). Composite wind speed profiles for wind events of varying peak intensity were calculated for the ERA5 wind data from the southern Chukchi Sea (Fig. 7A). A climatological analysis shows that wind events increase in frequency and intensity as summer progresses (Fig. 7B). For example, 14 m s^−1^ peak winds occurred an average of 0.5 times in June and rose to an average of 1.9 occurrences in September. We also characterize extreme years by identifying the maximum number of times that peak winds occurred in a given month across the dataset (Fig. 7B). Only one 18 m s^−1^ wind event was recorded in the dataset, occurring in September.

The thickness of the BBL varied throughout the DBO3 hydrographic climatology (Fig. 7C), averaging 18.6 ± 10.3 m with an average upper bound at 26.2 ± 10.5 m below the surface. A weak seasonal signal in the thickness of the BBL was observed over the summer months, with the upper boundary of the BBL becoming slightly shallower over the course of the summer.

The PWP simulations provided insight into the surface mixed layer depth under different wind conditions (Fig. 7D). These outputs can be compared to the BBL distribution (Fig. 7C) to determine the likelihood that cysts suspended within the BBL will have the opportunity to mix into surface waters during a given wind event or through repeated events (i.e., when the surface and bottom mixed layers overlap). A single 10 m s^−1^ peak wind event mixes down to ~12 m, at which depth ~7 % of BBLs in the hydrographic dataset would be accessed. By contrast, a single 16 m s^−1^ peak wind event deepens the mixed layer to ~22 m, at which depth ~40 % of BBLs in the hydrographic dataset would be accessed. Repeated wind events result in further deepening of the mixed layer to varying degrees based on the wind intensity. For instance, two 16 m s^−1^ wind events (with limited restratification between the events) would be sufficient to reach half of the BBLs in the climatology. In interpreting results, it should be noted that deeper mixing into the BBL would enhance the delivery of cysts into the surface mixed layer.

**Fig. 8.**
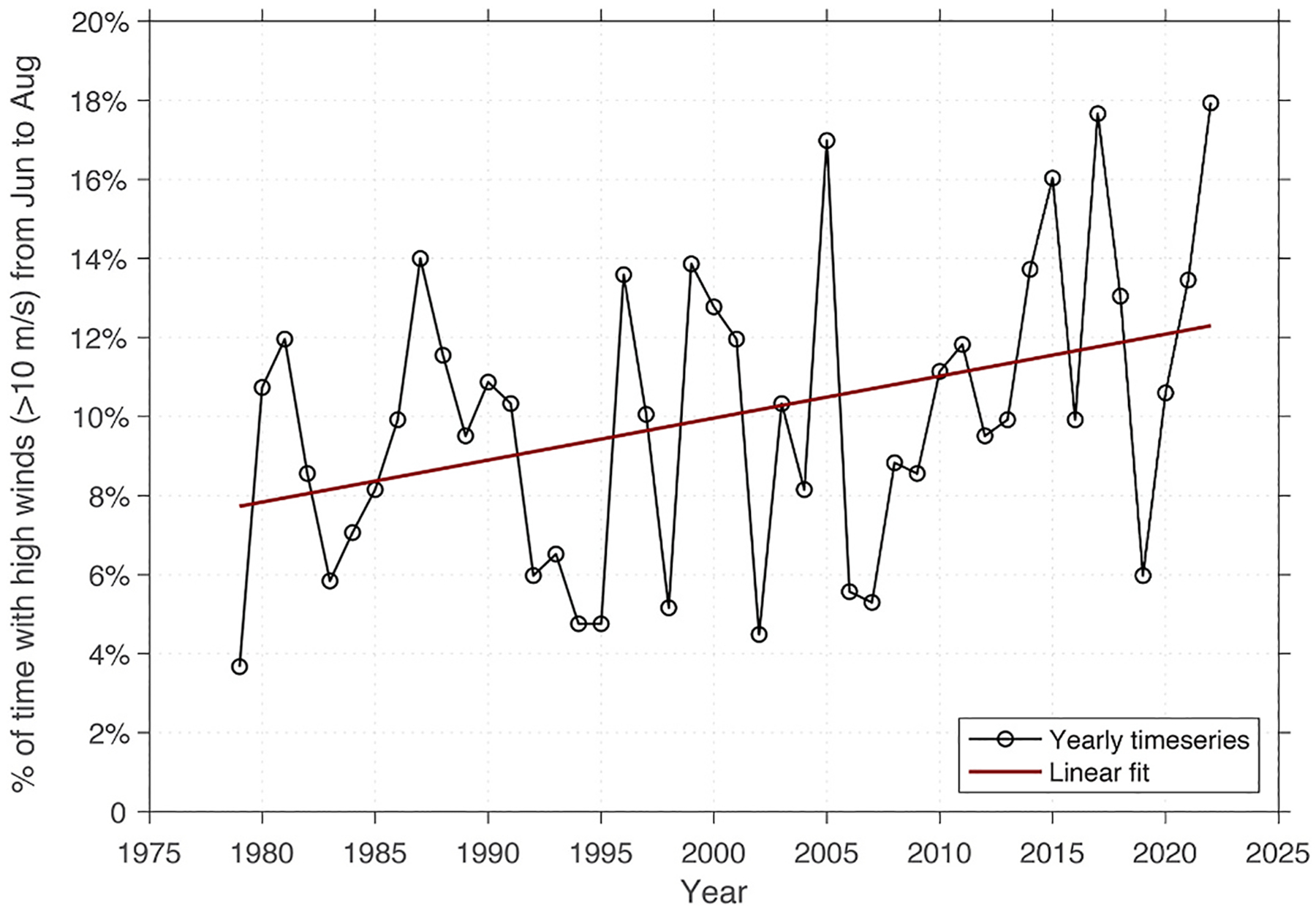
Proportion of time during the summer months (June–August) when wind speeds in the Ledyard Bay region (see box in Fig. 3A) are greater than 10 m s^−1^ based on ERA5 Reanalysis data from 1979 to 2022. Circles indicate annual values, line indicates the linear fit (p ≤ 0.05).

Although the seasonal wind data (Fig. 7B) indicate that high winds are not currently common in the summer months, historical analysis of wind speeds over the Ledyard Bay region (see box in Fig. 3A) reveals a significant trend towards stronger winds during the summer (June-–August) from 1979 to 2022 (Fig. 8). While wind conditions vary interannually, a linear fit (p < 0.05) indicated that the proportion of the time that the region experiences winds >10 m s^−1^ has increased from ~8 % to ~12 % over this period.

## 4. Discussion

Resuspension, transport, and deposition shape large-scale cyst distributions in both the benthos and the water column (Fig. 1), and these distributions will in turn influence regional bloom dynamics. Widespread presence of suspended *A. catenella* cysts was observed over the Chukchi shelf cyst bed (Fig. 3), with evidence of enhanced suspension at shallow, well-mixed stations. While suspended cyst abundance was higher in near-bottom waters and within the BBL, detection of cysts in the upper water column indicates that resuspension events can elevate cysts into surface waters (Fig. 4). Cyst flux time series revealed wind-driven cycles of resuspension and deposition through the fall and winter which varied over several orders of magnitude, as well as strong pulses of deposition in the late summer that are likely from terminating blooms (Fig. 5). Modeling of germination potential through the water column (Fig. 6) and wind-driven mixing (Fig. 7) provide further insight into the seasonality of resuspension as it relates to potential bloom initiation, highlighting the importance of interacting factors such as temperature, stratification, wind, and dormancy cycling in facilitating Arctic HABs.

### 4.1. Underlying drivers of cyst suspension

Wind is a key driver of cyst resuspension in the region, increasing current velocities and forming waves which enhance shear stress at the benthic interface thereby mobilizing cysts into the water column. The association between peak winds and cyst fluxes in the timeseries data demonstrates the importance of wind in modulating cyst suspension and deposition. During the fall 2020 survey, strong resuspension was observed at shallow well-mixed stations, evidenced by increased particle concentrations and elevated oxygen levels in near bottom waters. The association of suspended cysts with fresher waters is to be expected at shallow coastal stations where freshwater input is prevalent. However, the abundances of suspended cysts (max 15,800 cysts m^−3^) observed during the hydrographic survey were somewhat lower than expected given the extremely high concentrations of cysts in the Chukchi shelf cyst bed. In the Gulf of Maine, a region with benthic cyst densities roughly one order of magnitude lower, nearly half a million cysts m^−3^ have been reported in bottom waters (Pilskaln et al., 2014b), though cyst abundance in the water column was highly variable over time (Kirn et al., 2005). Tides may explain this discrepancy between systems; while the Gulf of Maine experiences strong semi-diurnal tidal currents that facilitate resuspension (Butman et al., 2014), there is no strong tidal forcing on the Chukchi shelf (Baumann et al., 2020), highlighting the importance of winds in driving suspension there. Moreover, our spatial survey represents a single snapshot, but observed seasonal and interannual variability in cyst fluxes points to the likelihood that the region experiences a dynamic distribution of cysts throughout the water column that can vary over several orders of magnitude through the year (Fig. 5).

Wind-driven resuspension on the Chukchi shelf is facilitated by its shallow water depth as well as the properties of the benthos. Bottom sediments range from fine sand inshore to silt and clay offshore (Pisareva et al., 2015), and are vulnerable to disturbance at relatively low shear stress. Butman et al. (2014) demonstrated that cysts in surface sediments in the Gulf of Maine are resuspended when bottom shear stress exceeds 0.1Pa, a condition that occurs frequently there during major storms or with strong tidal currents. Studies which estimate shear stress and erosion in the region of the Chukchi cyst bed would be highly informative; similar work on the Beaufort Shelf (Undzis et al., 2024 preprint) shows that wave stress dominates the shallow near-shore shelf while current stress prevails along the shelfbreak.

While this study focused on physical drivers of suspension, other activities have the potential to mobilize cysts into the water column. Walrus feeding leads to significant bioturbation in the Pacific Arctic region (up to 5000 km^2^ per year), enhancing sediment-water nutrient fluxes (Ray et al., 2006) and potentially also resuspending cysts. Foraging by gray whales also causes wide-scale resuspension and potential transport of sediment (Nelson et al., 1987). While there are currently no commercial fisheries in US waters of the Chukchi Sea (Christiansen et al., 2014), it should be kept in mind that trawling activity can result in dinoflagellate cyst resuspension (Brown et al., 2013; Giannakourou et al., 2005). The potential for mobilization of cysts by fishing gear should be considered in the future if fisheries expand into Arctic waters (Mueter et al., 2021).

### 4.2. Suspended cyst transport and deposition

Suspended dinoflagellate cysts can be transported by currents (Pilskaln et al., 2014a) advecting laterally over moderate to long distances (Zonneveld et al., 2022) and shaping the distribution of the underlying cyst bed (García-Moreiras et al., 2023). Cysts tend to accumulate in regions of low velocity, such as Ledyard Bay (Anderson et al., 2021), rather than in high-velocity areas such as the Bering Strait. Resuspension events would erode and transport away any cysts originally deposited in the strait, winnowing away the benthic population over time. For example, at a sinking rate of 0.1 mm s^−1^ (Aretxabaleta et al., 2014), a cyst mixed to mid-depths in the Bering Strait (~20 m above the seafloor) would take ~2.3 days to settle back to the benthos in the absence of further mixing. During this time, at velocities ranging from 13 to 35 cm s^−1^ based on monthly mean velocities from a Bering Strait mooring climatology (Woodgate, 2018), it would travel 26–70 km during its descent. Note that these calculations refer to cysts in a marine-snow type matrix; it is unknown how mucilaginous aggregations composed predominantly of cysts, previously observed in water samples collected in the Beaufort Sea (Supplementary Fig. 3), may influence settling rate and overall transport distances. In any case, multiple resuspension events in these generally northward flowing waters would gradually sweep cyst biomass from the Bering Strait region to the Chukchi shelf, where slower current speeds promote retention and accumulation in the benthos (Anderson et al., 2021).

This was observed most strikingly at the DBO2 mooring site (Fig. 5), where a dense pulse of cysts was deposited in August 2017. Given the high volume of cysts, late summer timing of the event, and relative lack of cyst detection in the traps leading up to these large pulses, we believe that this flux represents a cohort of newly formed cysts, presumably from a terminating bloom advecting northward through the Bering Strait (e.g. Fachon et al., 2025; Natsuike et al., 2017a). Over the course of the fall and winter, the fluxes of cysts gradually decreased at that site, with no strong new depositional pulses observed the following summer and relatively low fluxes in fall 2018 and winter 2019 compared to the year prior. The imbalance between annual integrated cyst fluxes and surrounding benthic cyst concentrations at the mooring sites also provides evidence of loss due to transport processes. Given the shallow depth and high velocity of currents passing through the Bering Strait (Woodgate, 2018), the most likely explanation for this disparity is loss of the underlying benthic population over time as a result of current and wave-driven resuspension and transport, although other loss processes such as germination, bioturbation, and death may also play a role.

Further northeast along the Chukchi shelf, the role of lateral transport in shaping the cyst bed is less clear. The offshore signal of resuspension observed on the Ledyard Bay transect appears to correspond with the Central Channel pathway, but no strong velocity correlation was detected during the spatial survey. More observations are needed to elucidate the role that Chukchi shelf pathways (Fig. 2) may play in transporting cysts, including whether the high velocities through Barrow Canyon (Watanabe et al., 2022) result in any transport off the shelf and into the Canada Basin.

Another factor that may shape patterns of distribution is the seasonal presence of sea ice in the region. Resting cysts of some dinoflagellate species are known to reside in sea ice (Buck et al., 1992; Kauko et al., 2018), and, although *A. catenella* is not known for this phenomenon, we have observed dense cyst aggregates in surface waters at the ice edge (Supplementary Fig. 3). Entrapment during ice formation, or during sediment entrainment (Eicken et al., 2005), and release during the breakup process may play a role in moving and depositing cysts around the region. Additionally, after freeze-up, the effect of wind-driven resuspension on the lower water column will be dampened by the presence of pack ice (Eidam et al., 2025).

### 4.3. Resuspension and germination potential

Resuspension, even for brief periods of time (Genovesi et al., 2009), can facilitate dinoflagellate cyst germination by exposing cysts to enhanced light, warmer temperatures, and oxygenation (Nehring, 1996). Furthermore, cysts that germinate in suspension benefit from nearer access to the euphotic zone, as germling mortality is significantly higher in dark conditions (Vahtera et al., 2014) and warmer surface waters allow for faster division rates and accumulation of vegetative cells (Lago et al., 2025). However, the effect of suspension upon cyst germination will be seasonal and dictated by interaction between temperature, stratification, wind strength, and dormancy cycling of *A. catenella*.

While *A. catenella* blooms generally start in the late spring and early summer at temperate latitudes (Ishikawa et al., 2014; McGillicuddy et al., 2005; Ralston et al., 2015; Rathaille and Raine, 2011), cooler temperatures in the Arctic shift the expected bloom window of this species into mid/late summer (Lago et al., 2025) with cells sometimes persisting into early fall (Natsuike et al., 2017a). During June in the southern Chukchi Sea, both bottom and surface temperatures are too cold for significant germination (Fig. 6B). As the summer progresses, temperatures warm throughout the water column and particularly at the surface, leading to a strong difference in germination potential between the BBL and upper waters. By contrast, our analyses did not reveal a strong difference in germination rates between the benthos and the BBL, although there is a small amount of enhancement resulting from resuspension. Cysts resuspended from the benthos can be mixed into the BBL, but stratification (Zhang et al., 2024) poses a barrier to accessing the warmer surface mixed layer.

What is the likelihood that winds will facilitate suspension of cysts into the surface mixed layer during the bloom season? Wind events increased in frequency and intensity over the course of the summer (Figs. 5 and 7B), and summer wind speeds in the region of the Ledyard Bay cyst bed also exhibited an increasing trend over time (Fig. 8). The PWP model results indicate that higher wind speeds create opportunities for the surface mixed layer to entrain waters from the BBL, potentially elevating cysts into surface waters. Based on historical data, typical wind conditions in July and August would only reach a small fraction of BBLs, but in extreme years up to half may be accessed (Fig. 7D). In September, wind conditions continue to escalate and temperatures remain warm and conducive to germination. However, our calculations of germination rates (Fig. 6) assume that the entire population of cysts is quiescent (able to germinate), and do not account for dormancy cycling (Brosnahan et al., 2020; Fischer et al., 2018). Prior work on populations from the Chukchi Sea indicate that the majority of the cyst population is dormant (unable to germinate) from September through December (Natsuike et al., 2017b). Therefore, although temperatures may remain favorable for germination into September as wind events become more intense, it is unlikely that resuspension would lead to significant *A. catenella* germination at this time as most of the population has entered dormancy. Still, resuspension of resting stages has been implicated as a driver of emerging fall diatom blooms (Fukai et al., 2025), and, as the region transitions to warmer summers with later freeze-up, persistence of productivity into the fall is becoming more common (Ardyna et al., 2014; Waga and Hirawake, 2020). Even in the absence of new *A. catenella* germination, a wind event in September may replenish nutrients to the mixed layer and prolong any blooms that are already in progress.

Overall, our analyses point to a narrow window in mid-to-late summer when resuspension may facilitate *A. catenella* bloom initiation. Under typical wind conditions, resuspension of cysts into surface waters remains unlikely, but the region is experiencing a shift towards more strong and frequent storms during all seasons (Sepp and Jaagus, 2011; Vermaire et al., 2013), and in Ledyard Bay wind speeds during the summer months are increasing (Fig. 8). These results highlight several important areas for further work. First, dormancy cycling of Arctic populations is not yet well characterized, but it is clear that the seasonal effect of resuspension will be highly sensitive to the fraction of the population that is quiescent. Future work should aim to constrain the effects of polar temperature regimes on dormancy cycling of *A. catenella* populations, as well as consider how climate change may influence these relationships. Second, our models of germination are based on temperature and light responses of cysts (Anderson et al., 2005; McGillicuddy et al., 2005), but do not account for the role of oxygen in queuing excystment (Anderson et al., 1987; Kremp, 2000). Oxygen availability varies broadly through the water column and within sediments (Cai and Sayles, 1996), and cysts in suspension are likely to experience significantly elevated oxygenation relative to their buried counterparts. The role of oxygen in germination should be incorporated into future studies which consider the contributions of resuspension to bloom initiation. Finally, our PWP modeling approach takes a top-down view of mixing but does not encompass the bottom-up processes that entrain cysts from the benthos into the BBL; further work could include wave models and estimates of orbital motion and shear stress at the benthic interface.

## 5. Conclusions

**Fig. 9.**
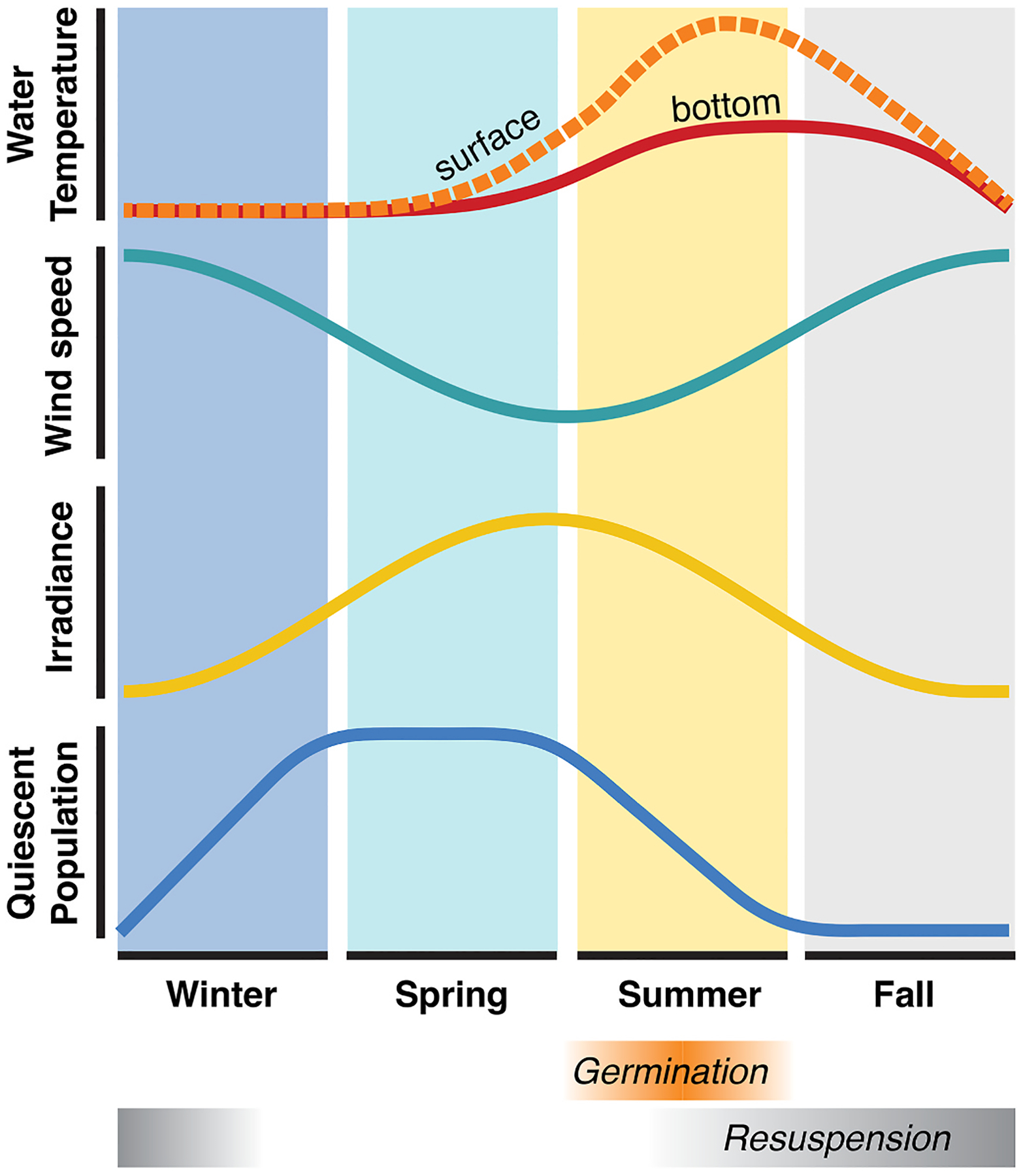
Conceptual diagram illustrating seasonality of germination drivers: surface and bottom water temperature, wind speed, irradiance (Dissing and Wendler, 1998), and proportion of cyst population that is quiescent (Natsuike et al., 2017b). The germination window extends through the summer, and the resuspension window begins in mid-summer peaking in the fall.

Through the spatiotemporal observations and model results compiled in this study, we can piece together an annual picture of *A. catenella* cyst resuspension on the Chukchi shelf (Fig. 9). In the fall and winter, storm and wind activity can suspend and transport cysts, redistributing any new deposition from the prior bloom season, although this effect is tempered by the seasonal presence of sea ice, which limits the effect of winds on the benthos (Eidam et al., 2025). During the winter, cold temperatures, low light levels, and population dormancy suppress cyst germination. Cysts begin to enter quiescence through the winter, with the population becoming fully quiescent in the spring (Natsuike et al., 2017b). However, spring temperatures remain insufficient throughout the water column to promote cyst germination, and although the daylength is becoming longer, decreasing wind speeds make resuspension less likely. Temperatures warm through the summer, increasing the potential for germination throughout the water column, particularly in July and August. While germination potential is significantly enhanced in surface waters relative to the benthos and benthic boundary layer, stratification and low winds limit access of the cysts to the surface mixed layer. Our PWP analysis reveals that under certain high-wind conditions, the surface waters may become accessible to cysts, particularly as the summer progresses and the frequency and intensity of wind events increases. This effect is counterbalanced by the waning quiescence of the cyst population through the summer (Natsuike et al., 2017b), highlighting the sensitivity of the system to temporal changes in any of these interacting drivers (temperature, winds, dormancy cycling; Fig. 9). In late summer, we see signs that active blooms are encysting and depositing new cysts, and increased wind speeds and associated resuspension are evident through the fall. However, despite lingering warm temperatures, population dormancy during the fall prevents new blooms from initiating.

Given the rapid ecosystem-scale changes that the Pacific Arctic region is experiencing (Grebmeier, 2012; Huntington et al., 2020), an understanding of HAB dynamics is essential in evaluating ecosystem health. It is clear from this study that cyst resuspension in the Pacific Arctic region is seasonal, highly variable through time, and has the potential to shape large-scale cyst distributions. As the strength and frequency of storm events in the Arctic increases across all seasons, as is expected with climate change (Sepp and Jaagus, 2011; Vermaire et al., 2013), resuspension events during the bloom season will become a more common occurrence. The shallow Chukchi shelf is easily influenced by even moderate wave and storm energy, making the dense cyst bed in the Ledyard Bay region vulnerable to disturbance. While the present effects of disturbance on this system may be limited, in a future warm and stormy Arctic cyst resuspension will have the potential to impact the timing and development of *A. catenella* blooms.

## Supplementary Material

MMC1

## Figures and Tables

**Table 1:** Spearman’s rank correlation coefficients (rho) between benthic cyst concentrations, near-bottom suspended cyst concentrations, suspended:benthic ratio and various spatial or environmental parameters.

	Suspended Cysts n = 77	Benthic Cysts n = 41	Suspended:Benthic n = 36
Latitude	−0.16	−0.28	−0.44*
Longitude	−0.36*	−0.77**	−0.26
Depth	−0.24*	−0.05	−0.50*
Temperature	0.37**	0.83**	0.18
Salinity	−0.09	−0.05	−0.37*
Oxygen	0.09	−0.04	0.34*
Fluorescence	0.40**	0.46*	0.57**
Beam Transmission (%)	−0.55**	−0.66**	−0.46*
Near-bottom Velocity	0.10	0.01	0.13

* indicates p < 0.05,

** indicates p < 0.001.

**Table 2:** Peak cyst fluxes, timing of peak cyst fluxes, and annual cyst flux for each sediment trap deployment. Average benthic cyst concentration (cysts cm^−3^ in the 0–3 cm layer) measured in summer following each sediment trap deployment calculated based on all sediment collections within 50 km of each trap site (Fachon and Anderson, 2021).

Trap Site	Sampling Period	Peak Flux (cysts cm^−2^ day^−1^)	Peak Flux Period	Annual Flux (cysts cm^−2^ year^−1^)	Mean benthic cyst concentration following trap deployment (cysts cm^−3^ ± SD)
DBO2 (N4)	6/26/2017 – 6/8/2018	23.52	8/12/2017 – 8/20/2017	1772	196 ± 164 (n = 4)
DBO2 (N4)	6/24/2018 – 6/27/2019	3.72	11/15/2018 – 12/17/2018	396	125 ± 81 (n = 3)
DBO3 (N6)	6/17/2017 – 6/8/2018	12.9	8/4/2017 – 8/12/2017	1078	399 ± 218 (n = 5)
DBO3 (N6)	6/24/2018 – 7/13/2019	6.17	11/7/2018 – 12/9/2018	356	94 ± 59 (n = 5)
DBO4 (CEO)	8/7/2018 – 7/30/2019	5.67	11/1/2018 – 11/15/2018	414	105 ± 88 (n = 7)

## Appendix A. Supplementary data

Supplementary data to this article can be found online at https://doi.org/10.1016/j.dsr2.2025.105567.
