# Supplementary material for: Spatiotemporal patterns and implications of suspended Alexandrium catenella cysts in the Pacific Arctic region
Source: Deep Sea Res 2 Top Stud Oceanogr. Author manuscript; Available in PMC 2026 Feb 4. (PMC12867128; doi:10.1016/j.dsr2.2025.105567)
Supplement: MMC1 [file NIHMS2126078-supplement-MMC1.pdf]

Supplementary Material for:

Spatiotemporal patterns and implications of suspended *Alexandrium catenella* cysts in the Pacific Arctic region

Evangeline Fachon[1,2*], Robert S. Pickart[3], Jie Huang[3], Catherine Lalande[4], Donald M. Anderson[1]
**corresponding author*

[1] Biology Department, Woods Hole Oceanographic Institution, Woods Hole, MA, USA
[2] Massachusetts Institute of Technology (MIT)-WHOI Joint Program in Oceanography/Applied Ocean Science & Engineering, Cambridge and Woods Hole, MA, USA
[3] Physical Oceanography Department, Woods Hole Oceanographic Institution, Woods Hole, MA USA
[4] Korea Polar Research Institute, Division of Ocean and Atmosphere Sciences, Incheon, South Korea

This PDF file includes:

- **Equations and parameters for germination model**
- **Supplementary Figure 1: Hydrographic profile distributions**
- **Supplementary Figure 2: Suspension prevalence in relation to key variables**
- **Supplementary Figure 3: Cyst aggregates detected in November 2018**

Equations and Parameters for Germination Model

Parameters for germination equations (Supplementary Table 1) were derived from curves experimentally fitted to Gulf of Maine cysts exposed to light and dark conditions (Anderson et al., 2005), with the exception of E_{lgt}, which was reported in McGillicuddy et al., 2005 (2.4 W m^{-2}).

Parameter	Definition	Units	Value (lgt/drk)
E_0	Surface irradiance	W m^{-2}	Supplementary Table 2
$E_{lgt/drk}$	Threshold light level for germ conditions	W m^{-2}	2.4 / 0.024
k	Diffuse attenuation coefficient	m^{-1}	0.2
$\alpha_{lgt/drk}$	Slope of linear temperature relationship	$(°C)^{-1}$	0.790 / 0.394
$\beta_{lgt/drk}$	Constant for tangent fit	Dimensionless	6.27 / 3.33
$G_{max(llgt/drk)}$	Maximum potential germination	% day^{-1}	8.72 / 4.26
$G_{min(lgt/drk)}$	Minimum potential germination	% day^{-1}	1.50 / 1.04

Supplementary Table 1. Parameters used for germination model (Anderson et al., 2005; McGillicuddy et al., 2005)

Percent germination was calculated based on temperature and irradiance, following the relationship defined in Anderson et al. 2005:

$$\text{when } E \geq E_{lgt} \qquad G(T,E) = G(T,E_{lgt})$$
$$\text{when } E \leq E_{drk} \qquad G(T,E) = G(T,E_{lgt})$$
$$\text{when } E_{lgt} > E > E_{drk} \qquad G(T,E) = G(T,E_{drk}) + (G(T,E_{lgt}) - G(T,E_{drk})) \times \frac{E - E_{drk}}{E_{lgt} - E_{drk}}$$

$$G(T,E_{lgt}) = \{G_{min\,(lgt)} + \frac{(G_{max\,(lgt)} - G_{min\,(lgt)})}{2} * [tanh(\alpha_{lgt}T - \beta_{lgt}) + 1]$$

$$G(T,E_{drk}) = \{G_{min\,(drk)} + \frac{(G_{max\,(drk)} - G_{min\,(drk)})}{2} * [tanh(\alpha_{drk}T - \beta_{drk}) + 1]$$

Light was attenuated across depths following the Bouguer-Beer-Lambert law (Kirk, 1977):

$$E = E_0 e^{-kz}$$

Where E is the irradiance at depth (z), E_0 is the surface irradiance, and k is the vertical attenuation coefficient. Surface irradiance for each month was acquired from (Dissing and Wendler, 1998) (Table 2), which presents a climatology of irradiance values for sites around Alaska. The climatology from Kotzebue Sound was selected for this analysis.

Month	Surface Irradiance (E_0) W m^{-2}
June	286
July	188
August	142
September	95

Supplementary Table 2. Monthly surface irradiance values from Dissing and Wendler 1998.

References:

Anderson, D.M., Stock, C.A., Keafer, B.A., Bronzino Nelson, A., Thompson, B., McGillicuddy, D.J., Keller, M., Matrai, P.A., Martin, J., 2005. Alexandrium fundyense cyst dynamics in the Gulf of Maine. Deep Sea Res. Part II Top. Stud. Oceanogr. 52, 2522–2542. https://doi.org/10.1016/j.dsr2.2005.06.014

Dissing, D., Wendler, G., 1998. Solar Radiation Climatology of Alaska. Theor. Appl. Climatol. 61, 161–175. https://doi.org/10.1007/s007040050061

Kirk, J., 1977. Attenuation of light in natural waters. Mar. Freshw. Res. 28, 497. https://doi.org/10.1071/MF9770497

McGillicuddy, D.J., Anderson, D.M., Lynch, D.R., Townsend, D.W., 2005. Mechanisms regulating large-scale seasonal fluctuations in Alexandrium fundyense populations in the Gulf of Maine: Results from a physical–biological model. Deep Sea Res. Part II Top. Stud. Oceanogr. 52, 2698–2714. https://doi.org/10.1016/j.dsr2.2005.06.021

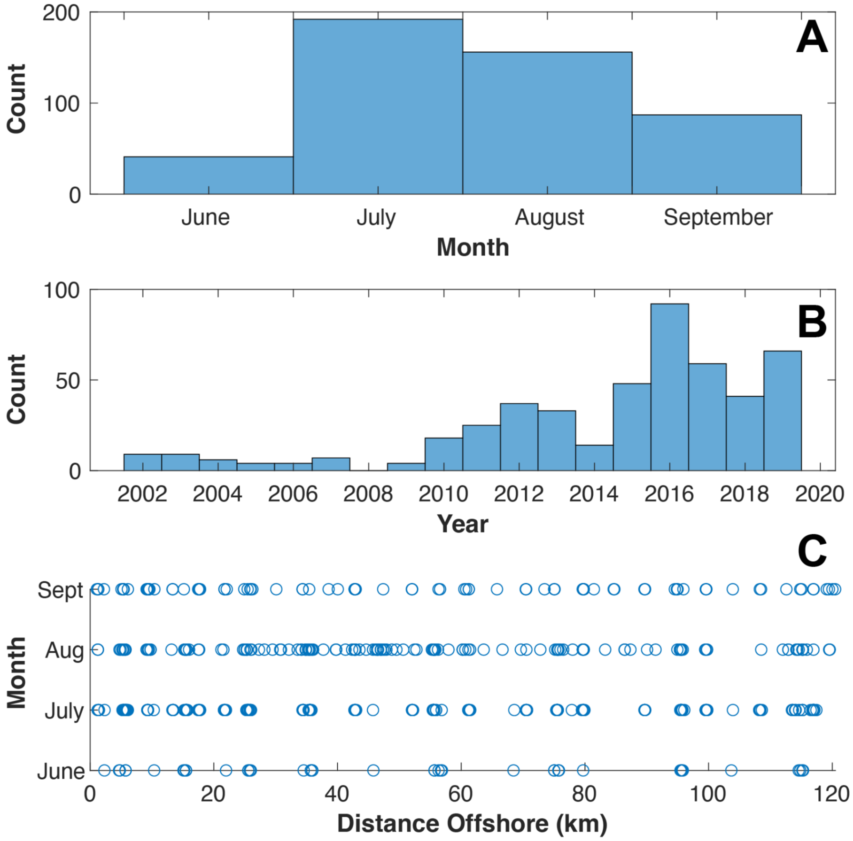

Supplementary Figure 1. (A) Monthly and (B) yearly distribution of hydrographic profiles from the DBO3 region climatology (Pickart et al., 2023) used for germination estimation and PWP mixing models. (C) Distribution of profiles along the DBO3 transect by month and distance offshore (km) from Point Hope.

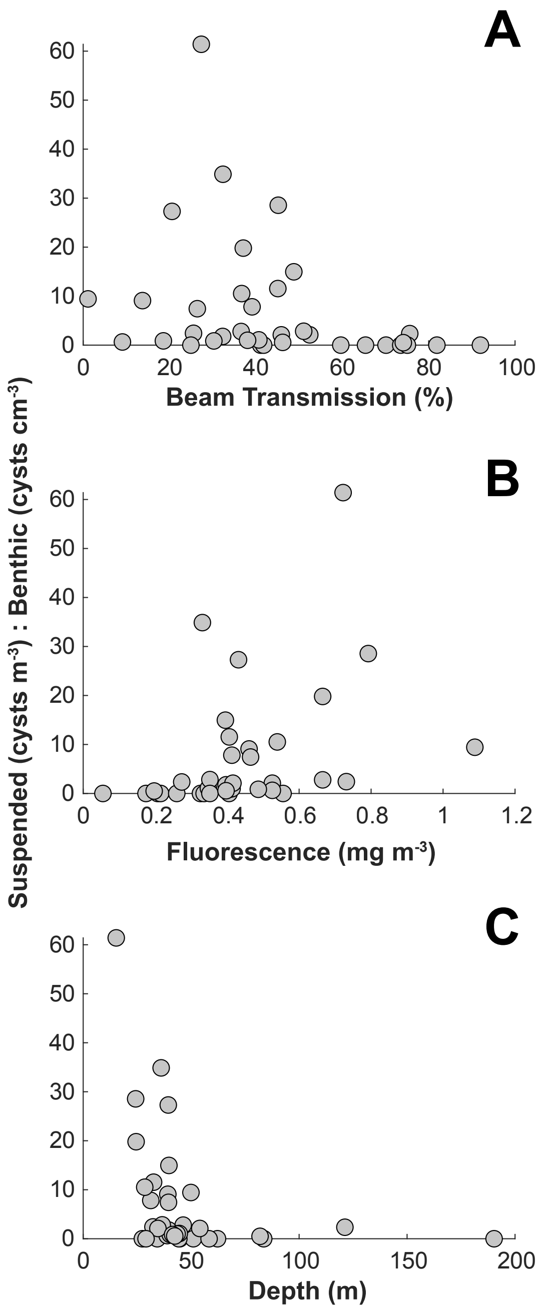

Supplementary Figure 2. Relationships between ratio of near-bottom suspended cysts (m^{-3}) to benthic cysts (cm^{-3}) and various co-measured environmental parameters. (A) Beam transmission (%), (B) Fluorescence ($mg\ m^{-3}$), and (C) Depth (m).

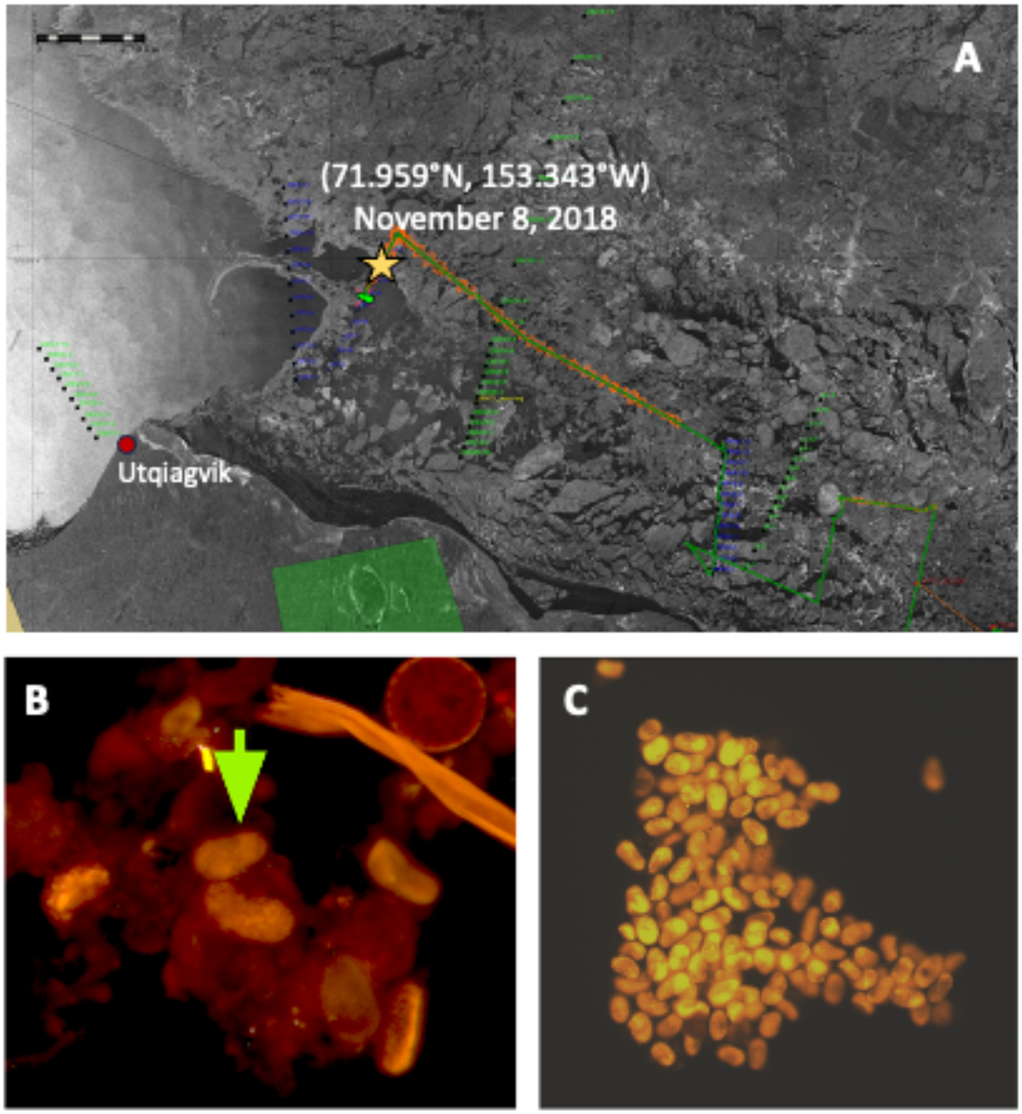

Supplementary Figure 3. Aggregates of cysts detected in near-surface waters at the ice edge in the Beaufort Sea, collected on November 8, 2018 by a CTD-mounted Niskin bottle. (A) Location of sample overlaid on ice imagery. (B-C) Cyst aggregates visualized under a Cy3 filter set.
